# Dietary Cholesterol Concentration and Duration Degrade Long-Term Memory of Classical Conditioning of the Rabbit's Nictitating Membrane Response

**DOI:** 10.1155/2012/732634

**Published:** 2012-04-11

**Authors:** Bernard G. Schreurs, Desheng Wang, Carrie A. Smith-Bell, Lauren B. Burhans, Roger Bell, Jimena Gonzalez-Joekes

**Affiliations:** ^1^Blanchette Rockefeller Neurosciences Institute, West Virginia University, Morgantown, WV 26506, USA; ^2^Department of Physiology and Pharmacology, West Virginia University, P.O. Box 9302, Morgantown, WV 26506, USA; ^3^Department of Neurobiology and Anatomy, West Virginia University, Morgantown, WV 26506, USA

## Abstract

A rabbit model of Alzheimer's disease based on feeding a cholesterol diet for eight weeks shows sixteen hallmarks of the disease, including learning and memory changes. Although we have shown 2% cholesterol and copper in water can retard learning, other studies show feeding dietary cholesterol before learning can improve acquisition whereas feeding cholesterol after learning can degrade long-term memory. We explored this issue by manipulating cholesterol concentration and duration following classical trace conditioning of the rabbit's nictitating membrane response and assessed conditioned responding after eight weeks on cholesterol. First, rabbits given trace classical conditioning followed by 0.5%, 1%, or 2% cholesterol for eight weeks showed body weight and serum cholesterol levels that were a function of dietary cholesterol. Although all concentrations of cholesterol showed some sign of retarding long-term memory, the level of memory retardation was correlated with serum cholesterol levels. Second, rabbits given trace conditioning followed by different durations of a 2% cholesterol diet combined with different durations of a 0% control diet for 8 weeks showed duration and timing of a 2% cholesterol diet were important in affecting recall. The data support the idea that dietary cholesterol may retard long-term memory.

## 1. Introduction

In rabbits fed 2% cholesterol for as little as eight weeks, there are as many as sixteen different indices of pathology that are similar to those seen in Alzheimer's Disease (AD) including intracellular and extracellular A*β*, breaches of the blood brain barrier, activation of microglia, apoptosis, increased levels of Apolipoprotein E, phosphorylated tau protein, changes in the cerebrovasculature, and increases in ventricular volume [1–12]. Coinciding with these changes in brain pathology, there have been reports of detrimental effects on learning [6, 13–16]. Nevertheless, research with this and other animal models suggests that modifying dietary cholesterol can in many cases *improve* learning and memory. For instance, increasing cholesterol in young DBA/2 mutant mice improves performance on the Morris water maze—a spatial learning task that is normally impaired in this mutant [17, 18]. Feeding cholesterol to young, normal rats also improves performance on the Morris water maze [19]. Feeding cholesterol to rats that are either deficient in cholesterol or have cholesterol synthesis blocked reverses problems with learning and memory [20–23]. We have also shown that feeding rabbits cholesterol can facilitate classical conditioning of the nictitating membrane response (NMR) and heart rate [24–26].

Despite the data on cholesterol's ability to facilitate learning, we have shown more recently that a diet of 2% cholesterol can have detrimental effects on the long-term memory of classical conditioning of the rabbit NMR [27]. There is significant precedent for these effects in humans where high cholesterol has been shown to have a negative impact on cognition [28–31]. Interestingly, although significant, the detrimental effects on long-term memory for classical conditioning of the rabbit NMR we have seen following a cholesterol diet [27] were more subtle and transitory than cholesterol's effects on acquisition of the classical conditioned NMR [24–26].

The purpose of the present experiments was to determine whether the effects of cholesterol on the memory of acquired classical conditioning of the rabbit NMR could be replicated and whether the detrimental effects were affected by the concentration of cholesterol (Experiment  1) or the duration and timing of cholesterol (Experiment  2).

## 2. Experiment  1

The manipulation of dietary cholesterol concentration has been shown to have direct effects on serum cholesterol levels [32]. A previous study in our laboratory by Darwish and colleagues gave rabbits trace conditioning of the NMR for 10 days using a brief tone conditioned stimulus (CS) paired with an air puff unconditioned stimulus (US) with a 250 ms trace interval and then fed them cholesterol for eight weeks before examining recall using tone-alone trials over the course of ten days of extinction. We found that responding to the tone in rabbits fed cholesterol for eight weeks was significantly lower than rabbits fed normal chow [27]. In the present experiment, we fed each of three groups of rabbits with a different concentration of cholesterol (0.5, 1, or 2%) following classical conditioning of the rabbit NMR and compared them to a normal chow control group (0% cholesterol) in an effort to replicate the original observation and determine whether cholesterol effects on memory were a function of cholesterol concentration.

## 3. Methods and Materials

### 3.1. Subjects

Thirty six, three-month-old, male, New Zealand white rabbits (*Oryctolagus cuniculus*) supplied by Myrtle's Rabbitry (Thompsons Station, TN) weighed approximately 2.0–2.2 kg upon arrival. Animals were housed in individual cages, given 1 cup of food daily, free access to ultra-pure water (18 MΩ, Millipore Academic), and maintained on a 12-hour light/dark cycle. Rabbits were assigned to four groups (*n*′s = 9) each with a different concentration of cholesterol in their food: 0%, 0.5%, 1%, or 2%.

Cholesterol-fed rabbits received 0.5%, 1%, or 2% cholesterol incorporated into Purina rabbit chow (Purina 5321; Dyets Inc., Bethlehem, PA) and control rabbits (0% cholesterol) received normal Purina rabbit chow (Purina 5321, 0% added cholesterol) for 8 weeks following classical conditioning. Previous reports have shown that rabbits fed a cholesterol diet for eight weeks have serum cholesterol levels that reflect the concentration of cholesterol in the diet and may exceed 1,800 mg/dL if fed a 2% cholesterol diet [32, 33] compared to rabbits fed normal chow that have levels of less than 40 mg/dL [9, 34–36]. Rabbits were maintained in accordance with guidelines issued by the National Institutes of Health and the research was approved by the West Virginia University Animal Care and Use Committee.

### 3.2. Apparatus

The apparatus has been detailed by Schreurs and Alkon [37] who modeled their apparatus after those described by Gormezano [37–39]. Each rabbit was restrained in a Plexiglas box and trained in a sound-attenuating, ventilated chamber (Coulbourn Instruments, Allentown, PA; Model E10-20). A stimulus panel containing a speaker and a house light (10-W, 120-V incandescent lamp) was mounted at a 45° angle, 15 cm anterior to, and 15 cm above the subject's head. An ambient noise level of 65 dB was provided by an exhaust fan. A programmable air pressure delivery system (Model ER-3000, Tescom Corp., Elk River, MN) was used to deliver a puff of air through a tube (1 mm internal diameter) positioned 5 mm from and perpendicular to the center of the cornea.

 Details of transducing nictitating membrane (NM) movements have been reported previously [37, 40]. A 1 mm hook connected to an L-shaped lever containing a freely moving ball and socket joint was attached to a 6-0 nylon loop sutured into, but not through, the NM. The other end of the lever was attached to a potentiometer (Novotechnik US Inc., Southborough, MA; Model P2201) that, in turn, was connected to a 12-bit analog-to-digital converter (5 ms sampling rate; 0.05 mm resolution). Individual analog-to-digital outputs were stored on a trial-by-trial basis for subsequent analysis. Data collection, analysis, and stimulus delivery were accomplished using a LabVIEW system (National Instruments, Austin, TX).

### 3.3. Procedure

All rabbits received one day of adaptation, ten daily sessions of tone and air puff trace conditioning, and following an eight-week diet of 0%, 0.5%, 1% or 2% cholesterol, ten days of tone-alone extinction. The cholesterol diet was continued during the ten days of tone-alone extinction. The adaptation session allowed rabbits to habituate to restraint and the training chambers. Trace conditioning was used to establish anticipatory conditioned responses (CRs) and, on the basis of their performance, rabbits were assigned to treatment conditions to equate the level of CR acquisition in each group. Trace conditioning engages a number of brain regions including the hippocampus and cortex [41]—areas in the rabbit brain where beta amyloid deposits occur following a high cholesterol diet [42]. CS-alone extinction serves to assess the effects of the cholesterol diet on retention of classical conditioning and also engages the hippocampus and cortex [43]—two areas particularly affected by Alzheimer's Disease [44].

On adaptation day, the rabbits were prepared for recording of NM movement and then adapted to the training chambers for the length of time of subsequent training sessions (60 min). Each of the ten paired trace conditioning sessions consisted of 60 presentations of a 250 ms, 1 KHz, 82 dB, tone CS that was followed by a 250 ms trace interval and then a 100 ms, 4 psi air puff US (i.e., 500 ms interstimulus interval). Each of the ten days of tone-alone extinction consisted of 60 presentations of a 250 ms, 1 KHz, 82 dB, tone CS. Stimulus presentations were delivered, on average, every 60 s (50–70 s range).

An NM CR was defined as any extension of the NM exceeding 0.5 mm that was initiated after CS onset but prior to US onset on paired trials and the point at which the US would have occurred on tone-alone extinction trials.

### 3.4. Statistics

Data were analyzed by analysis of variance and, where appropriate, by followup contrast of the means. Where possible, orthogonal contrasts were used to provide additional power to detect differences between groups. This added power to detect differences is derived from the ability of orthogonal contrasts to completely partition the sum of squares. The level of significance was set at *P* < .05.

### 3.5. Serum Cholesterol Levels

Total serum cholesterol levels from all but one of the rabbits in the 1% group were assessed at the end of the experiment using a colorimetric kit (BioAssay Systems, ECCH-100) following the manufacturer's instructions. Following anesthesia, blood was collected directly from the heart into lavender top (EDTA additive) tubes. Blood was centrifuged and serum was separated and frozen for subsequent analysis with the colorimetric kit.

## 4. Results

### 4.1. Body Weight

**Figure 1:**
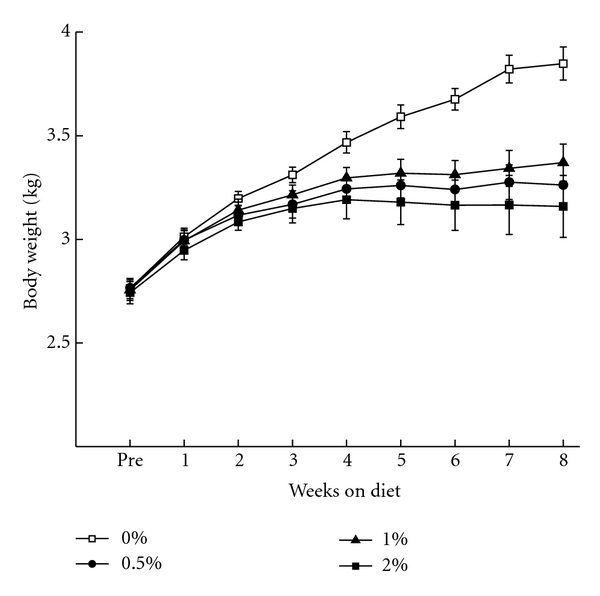
Mean (±SEM) body weight for rabbits in the 0%, 0.5%, 1%, and 2% cholesterol groups upon arrival (Pre) and across the eight weeks of the cholesterol diet.

Figure 1 shows mean body weights of rabbits on arrival (Pre) and over the course of eight weeks on a 0%, 0.5%, 1%, and 2% cholesterol diet and indicates that after eight weeks on the diet, rabbits on the 0.5%, 1%, and 2% cholesterol diets had gained less weight (2.82 kg) than the 0% cholesterol control group (3.44 kg). The lower weight gain in cholesterol-fed rabbits reflects the increasing pathology induced by cholesterol including inflammation, atherosclerosis, and hepatotoxicity [45–49]. Analysis of variance (ANOVA) confirmed a significant increase in weight as a functions of weeks on the diet (*F*(8, 256) = 111.83, *P* < .001) and a significant difference in weight between the groups (*F*(3, 32) = 6.16, *P* < .01). A significant interaction of group and weeks suggested that more weight was gained by the 0% than the cholesterol groups (*F*(24, 256) = 7.21, *P* < .001). This was confirmed by an orthogonal post-hoc comparison of the 0% cholesterol group against the other concentration groups which was significant from Week 4 through Week 8 (*F*′s(1,32) > 4.70, *P*′s < .05). There were no differences in the weights of rabbits on the 0.5%, 1%, and 2% cholesterol diets.

### 4.2. Serum Cholesterol

**Figure 2:**
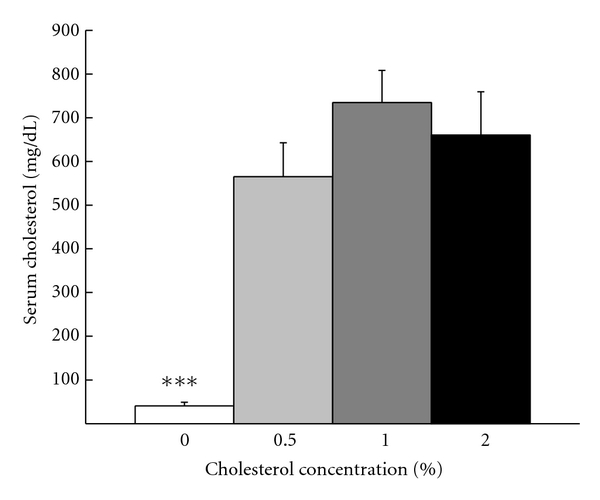
Mean total serum cholesterol levels at euthanasia for rabbits in the 0%, 0.5%, 1%, and 2% cholesterol groups. ***P < .001.

Figure 2 shows that at the time of euthanasia, rabbits on the 0.5% (565 mg/dL), 1% (735 mg/dL), and 2% (660 mg/dL) cholesterol diets had higher mean levels of serum cholesterol than the 0% control diet (41 mg/dL). Analysis of variance revealed a significant effect of cholesterol concentration (*F*(3,18) = 14.62, *P* < .001) and the same orthogonal post-hoc comparison used for body weight confirmed the lower serum cholesterol concentration in the 0% cholesterol group than the 0.5%, 1% and 2% groups (*F*(1,31) = 60.22, *P* < .001). There were no other significant differences in serum cholesterol concentration between the groups 0.5%, 1%, and 2%.

### 4.3. Behavior

#### 4.3.1. Classical Conditioning

**Figure 3:**
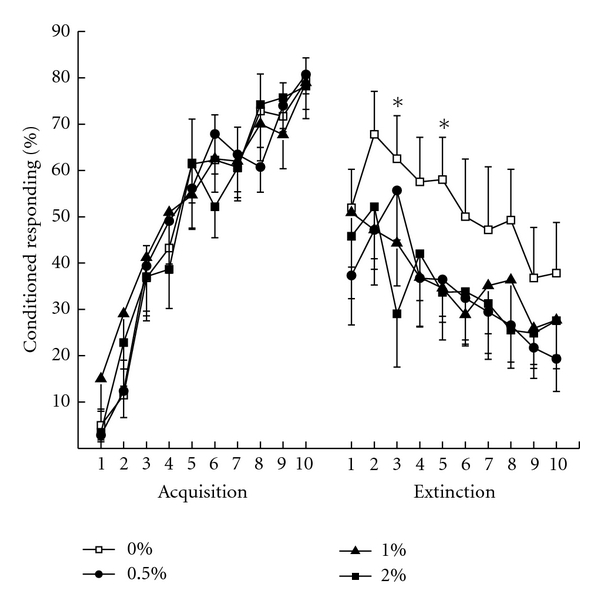
Mean percent conditioned responding during 10 days of Acquisition and 10 days of tone-alone Extinction separated by 8 weeks on a diet of 0%,  .5%, 1%, or 2% cholesterol added to normal rabbit chow. *P < .05.

Figure 3 shows mean percent CRs for rabbits in the four cholesterol concentration groups across the ten days of trace conditioning and the ten days of tone-alone extinction. The figure shows very comparable levels of CR acquisition across the ten days of trace conditioning (Acquisition) and ANOVA yielded a main effect of days of trace acquisition (*F*(9, 288) = 85.40, *P* < .001) but no other effects (*F*′s < 1). Following eight weeks on their respective diets, all rabbits showed a significant drop in the level of CRs from terminal levels during acquisition to the first day of CS-alone extinction (Extinction). Rabbit CR levels continued to decline across the remaining days of extinction with rabbits in the 0% group showing somewhat higher levels of responding than the 0.5%, 1%, and 2% groups which did not appear to differ from each other. An analysis of percent CRs comparing the last day of acquisition to the first day of CS-alone extinction yielded significant effect of extinction (*F*(1,32) = 32.16, *P* < .001) but no effects of group (*F*′s < 1). Analysis of percent CRs during tone-alone extinction yielded a significant effect of days of extinction (*F*(8, 288) = 7.95, *P* < .001) but no overall effect of group. Given the similar effects of 0.5%, 1%, and 2% cholesterol on body weight and serum cholesterol concentrations, we reasoned there may have been a similar overall effect of 0.5%, 1%, and 2% cholesterol on responding during tone-alone extinction as suggested by Figure 3. Consequently, we conducted an orthogonal comparison of group means across the days of extinction and found an overall difference between the level of CRs of the 0% cholesterol group and the remaining cholesterol concentration groups (*F*(1,34) = 4.02, *P* = .05) which was localized to significant differences on Days 3 and 5 of extinction (*F*′s > 4.36, *P*′s < .05).

#### 4.3.2. Serum Cholesterol and Extinction

An analysis of mean percent CRs during extinction and serum cholesterol levels found a significant negative correlation (*r* = −0.36, *P* = .016) suggesting that as serum concentration increased, the mean level of responding during extinction was lower.

## 5. Discussion

The overall findings of the present experiment were that feeding rabbits a diet high in cholesterol for eight weeks, regardless of whether the cholesterol concentration was 0.5%, 1%, or 2%, reduced weight gain, increased serum cholesterol, and, consistent with a previous finding [27], reduced retention of classical conditioning of the rabbit NMR. As with the previous finding by Darwish and colleagues, the present effect was subtle and this subtlety may be attributable, in part, to the inherent variability in extinction data with the rabbit NMR preparation [27, 50, 51] as can be seen in Figure 3 if one compares the relatively low variability in terminal levels of responding during acquisition (Day 10) to the levels of responding during the ten days of extinction. Another reason for the subtle nature of the differences between cholesterol-fed rabbits and controls was the relatively modest increases in serum cholesterol shown by rabbits in the 1% and 2% groups in the present experiment. In a number of other studies where a 2% cholesterol diet has been administered, serum cholesterol increased to levels in excess of 1,800 mg/dL [33] and although one rabbit in the present study had serum cholesterol levels in excess of 1,000 mg/dL, the mean level was only 660 mg/dL which was no different from the serum cholesterol level in rabbits given 0.5% or 1% cholesterol. If serum cholesterol levels were low compared to other studies, it follows that other consequences of cholesterol including the levels of sixteen different indices of pathology previously reported [6, 11, 12] might also have been lower and, as a result, reduced potential behavioral differences. Nevertheless, the effects of cholesterol were sufficient to affect recall of a previously learned task [27] and the level of that recall was a function of the concentration of cholesterol in the blood.

## 6. Experiment  2

One of the interesting aspects of feeding rabbits a cholesterol diet is that once the cholesterol is stopped there is a considerable delay in the regression of cholesterol's effects including changes in body weight, serum cholesterol levels, and aortic plaques [33, 52–55]. This delay in regression can occur after just six weeks on a 2% cholesterol diet [33]. Another interesting aspect of feeding rabbits cholesterol is that significant pathological changes can begin to occur in a number of indices including inflammation and microvascular changes almost immediately and be fully developed in as little as four weeks [56, 57]. The purpose of Experiment  2 was to determine the effects of manipulating the duration (0, 4 or 6 weeks) and timing of a 2% cholesterol diet on retention of the classically conditioned rabbit NMR.

### 6.1. Subjects

A total of fifty four, three-month-old, male, New Zealand white rabbits (*Oryctolagus cuniculus*) supplied by Myrtle's Rabbitry (Thompsons Station, TN) weighed approximately 2.0–2.2 kg upon arrival. Animals were housed in individual cages, given 1 cup of food daily, free access to ultra-pure water (18 MΩ, Millipore Academic), and maintained on a 12-hour light/dark cycle. Rabbits were assigned to five groups in which the duration and sequence of a 2% cholesterol diet was manipulated. Specifically, rabbits were fed 2% cholesterol for zero, four, or six weeks, and half the rabbits were fed the 2% cholesterol followed by normal Purina chow and the other half were fed normal Purina chow followed by the 2% cholesterol. Groups were designated 6C2P, 2P6C, 4C4P, 4P4C, and 0C8P where the letter “C” represents 2% cholesterol and the letter “P” represents normal Purina chow (0% cholesterol), the letter sequence indicates the order of the diets, and the number in each group designation represents the number of weeks on each diet.

Unless otherwise stated, the apparatus and procedures where the same as those used in Experiment  1.

An NM CR was defined as any extension of the NM exceeding 0.5 mm that was initiated after CS onset but prior to US onset on paired trials and the end of the observation interval on tone-alone extinction trials.

### 6.2. Serum Cholesterol Levels

Total serum cholesterol levels from all but three of the rabbits in the 6C2P group were assessed at the end of the experiment using a colorimetric kit as described for Experiment  1.

## 7. Results

### 7.1. Body Weight

**Figure 4:**
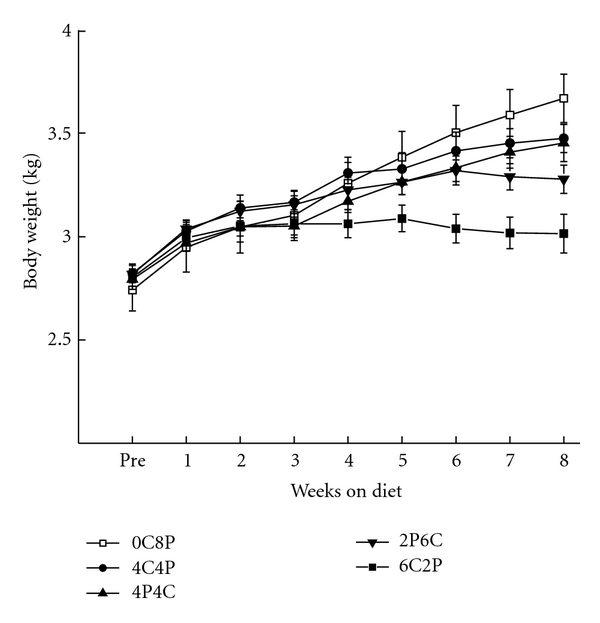
Mean (±SEM) body weight for rabbits upon arrival (Pre) and across the eight weeks of their respective diets in the 6CP2, 2PC6, 4C4P, 4P4C, and 0C8P groups where the numbers in the group designation refer to the number of weeks out of eight the rabbits were on either a 2% cholesterol diet (C) or on normal Purina rabbit chow (P). The order of the letters and refers to order in which rabbits received their respective diets.

Figure 4 shows mean rabbit body weights were a function of the duration and order of a 2% cholesterol diet. As in Experiment 1, rabbits on the 2% cholesterol diet had lower body weights than the normal Purina chow control rabbits. In addition, the differences in body weight were a function of duration on the diet with rabbits on the 2% cholesterol for six-weeks (6C2P and 2P6C) gaining the least weight and those fed the six-week 2% cholesterol diet before the normal Purina chow diet (6C2P) gaining less weight than those on the six-week 2% cholesterol diet after the normal chow (2P6C). An ANOVA revealed a significant main effect of weeks on the diet (*F*(8,392) = 167.82, *P* < .001) and an interaction of cholesterol duration and weeks on the diet (*F*(32,392) = 9.96, *P* < .001). Post-hoc comparisons of the interaction of groups and weeks confirmed that the normal Purina chow controls were heavier than the 2% cholesterol groups by Weeks 6, 7, and 8 (*P*′s < .05). The normal Purina chow controls were no different from the four-week cholesterol groups (4C4P, 4P4C, *P*′s > .06) but were heavier than the six-week cholesterol groups (6C2P, 2P6C) by Weeks 5, 6, 7, and 8 (*P*′s < .05). The rabbits fed 2% cholesterol for six weeks before two weeks on normal chow gained less weight than those fed 2% cholesterol for six weeks after two weeks on normal Purina chow on Weeks 6, 7, and 8 (*P*′s < .05). The data confirm that the longer the rabbits were on the diet the less weight they gained and that being on the diet had residual effects on weight gain even after a normal chow diet (0% cholesterol) was restored [33, 52].

### 7.2. Serum Cholesterol

**Figure 5:**
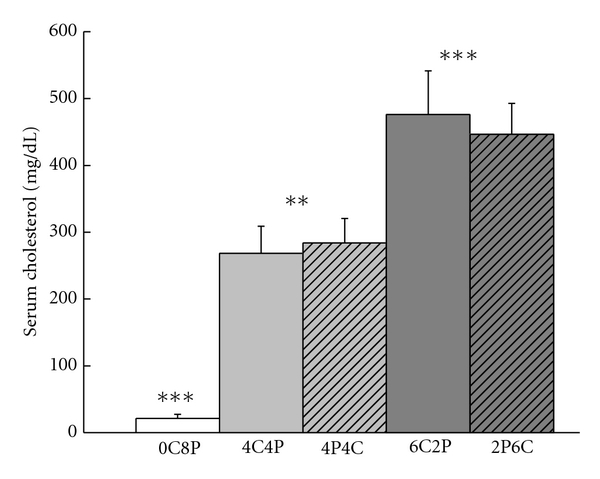
Mean total serum cholesterol levels at euthanasia for rabbits in the 6CP2, 2PC6, 4C4P, 4P4C, and 0C8P groups. **P < .01, ***P < .001.

Figure 5 shows that at the time of euthanasia, the mean levels of serum cholesterol were a function of the duration of the cholesterol diet regardless of whether the diet occurred before or after the Purina chow control diet. There were clearly no differences in mean serum cholesterol levels as a function of the order of the diet for either duration. Analysis of variance revealed a significant effect of cholesterol duration (*F*(4,46) = 19.90, *P* < .001) and a post-hoc comparison confirmed a lower serum cholesterol concentration in Purina chow control group than the cholesterol duration groups (*F*(1,46) = 52.12, *P* < .001). Postcomparisons also showed that mean serum cholesterol levels in the six week groups (6C2P and 2P6C) were significantly higher than the four week groups (4C4P and 4C4P) (*F*(1,46) = 20.83, *P* < .001) that were, in turn, significantly higher than controls (*F*(1,46) = 11.02, *P* < .01). There were no effects of the order of the diet on mean serum cholesterol for either the four-week or six-week groups (*F*′s < 1) suggesting that once serum cholesterol levels were elevated by the cholesterol diet they remained elevated when rabbits were placed back on normal Purina chow even for as long as more than five weeks in the case of 4C4P (four weeks plus ten days of extinction).

### 7.3. Behavior

#### 7.3.1. Classical Conditioning

**Figure 6:**
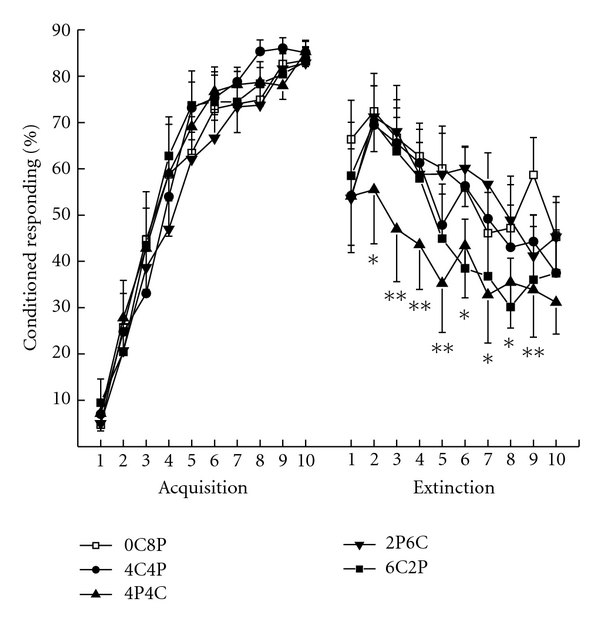
Mean percent conditioned responding during 10 days of Acquisition and 10 days of tone-alone Extinction separated by 8 weeks on a specific diet. The 6CP2, 2PC6, 4C4P, 4P4C, and 0C8P group designations refer to the number of weeks out of eight the rabbits were on either a 2% cholesterol diet (C) or on normal Purina rabbit chow (P). The order of the letters and refers to order in which rabbits received their respective diets. *P < .05, **P < .01.

Figure 6 shows mean percent CRs for rabbits in the four 2% cholesterol duration groups and the normal Purina chow control group across the ten days of trace conditioning and the ten days of tone-alone extinction. The figure shows very comparable levels of CR acquisition across the ten days of CS-US pairings and ANOVA yielded a main effect of days of trace acquisition (*F*(9, 441) = 180.20, *P* < .001) but no other effects (*F*′s < 1). Following eight weeks on their respective diets, all rabbits showed a significant drop in CRs from terminal levels of acquisition to the first day of CS-alone extinction. Rabbit CRs continued to decline across the remaining days of extinction with considerable variability from day to day and rabbits in the 4P4C group showed lower CR levels particularly compared to those in the 0C8P normal Purina chow control group.

An analysis of percent responding comparing the last day of acquisition to the first day of CS-alone extinction yielded a significant main effect of extinction (*F*(1,49) = 38.10, *P* < .001) but no effects of groups (*F*′s < 1). Analysis of percent responding during tone-alone extinction yielded a significant main effect of groups (*F*(4,49) = 2.94, *P* < .05) attributable to the lower level of responding in Group 4P4C, and a significant effect of days of extinction (*F*(9,441) = 15.97, *P* < .001). A post-hoc comparison of Groups 4P4C and 0C8P across extinction revealed significant differences on all but Days 1 and 10 (*F*′s  (1,49) > 4.81, *P*′s < .05).

#### 7.3.2. Serum Cholesterol and Extinction

An analysis of mean terminal levels of percent CRs during extinction and serum cholesterol levels found a significant negative correlation in Group 4P4C (*r* = −0.55, *P* = .038) but no other significant correlations for groups or time points.

## 8. Discussion

The principal finding of the current experiment was that as little as four weeks of 2% cholesterol can significantly reduce responding during extinction of a classical conditioned rabbit NMR. This was only the case if the cholesterol diet occurred for four weeks before the ten days of tone-alone extinction because feeding 2% cholesterol for four weeks immediately after acquisition of the classically conditioned response did not significantly affect responding during subsequent extinction. This recency effect of a 2% cholesterol diet did not occur if the diet was initiated six weeks before the ten days of tone-alone extinction. A second finding of the current experiment was the effects of different durations of the diet on rabbit body weight. Despite the deleterious effect of four weeks of cholesterol on responding during extinction, there was no significant reduction in rabbit body weight compared to normal chow control rabbits as a result of the diet. In contrast, six weeks of 2% cholesterol reduced body weight gain but did not have a significant effect on responding during extinction. A third finding of the experiment was the effect of cholesterol duration on serum cholesterol levels and the residual effects of the cholesterol diet even after the rabbits had been returned to a normal rabbit chow control diet (0% cholesterol). That is, once serum cholesterol levels became elevated as a result of the cholesterol diet, they remained elevated despite a return to the control diet [33].

One of the most important aspects of learning and forgetting a specific memory is the context in which learning takes place and whether recall takes place in the same or a different context [58–61]. Although context usually refers to a specific place such as the training chamber, it can also refer to the internal state of the animal [62–65]. For example, drug states and even illness can serve as very effective cues in a learning task [66–69]. Another aspect of learning and memory is the need for a period of consolidation and the transfer of memories during and after consolidation from one area of the brain (e.g., hippocampus) to others areas (e.g., prefrontal cortex) [59, 70–73]. A final aspect of learning and memory concerns the reactivation of memories that allows for reconsolidation but makes memories labile and prone to interference or forgetting [73–77]. The results of the present experiment may touch upon each of these different aspects of learning and memory. For example, it is possible that exposure to cholesterol that raises serum cholesterol levels and resultant brain changes [78], and hence the internal context of a previously trained rabbit at a critical point in the consolidation and transfer of memories to sites of permanent storage, may interfere with recalling that memory. There is some evidence that rabbits can remember classical conditioning of the NMR almost perfectly one month after it has been acquired but that the memory begins to fade slowly beyond that point in time [50, 51]. Some of the rabbits tested during extinction in these long-term memory experiments displayed memory reactivation and reconsolidation as a function of repeated days of tone-alone extinction because responding increased significantly after the first day of extinction [50, 51]. There is at least a suggestion that a similar reactivation appears to take place for a number of groups in the present experiment (as well as in Experiment  1) where response levels increase rather than decrease from Day 1 to Day 2 of extinction. The only group that shows no such increase in responding to the tone was group 4P4C. One could imagine that instituting a cholesterol diet one month after acquisition may have coincided with a critical period in consolidation and transfer of the memory from one brain site to another. The cholesterol diet may also have begun to change the internal context of the rabbit which continued to change up to and beyond the point of tone-alone extinction. This would not have occurred in the 2P6C group because the internal context would have been changed sufficiently before the onset of the critical period of consolidation and memory transfer. It would not have happened in Group 6C2P because the change in internal context would have begun immediately and, as the change in body weight suggests, would have outlasted the shift back to the normal Purina chow control diet.

## 9. General Discussion

The major finding of the present experiments was that dietary cholesterol may retard long-term memory. This confirms a previous study from our laboratory but, as already noted, the effects are not as robust as the effects of cholesterol on acquisition of a task.

The subtlety and complexity of the current findings may well reflect the complexity of forgetting and recall itself. There is considerable debate about the nature and anatomical locus of extinction [79, 80]. On the one hand, extinction has been proposed to involve new learning. On the other hand, it has been suggested that extinction involves erasure or unlearning of an old association [81–83]. In the case of the rabbit NMR, does the rabbit forget that tone signaled air puff or does it learn that tone no longer signals air puff? Evidence from previous studies of long-term memory for classical conditioning of the rabbit NMR provides evidence for both views [50, 51]. Rabbits given tone-alone extinction trials one, three, six, and even nine months after initial acquisition showed levels of responding to the tone that were a direct function of the time between acquisition and extinction supporting the notion that they had forgotten tone-predicted air puff. In some cases, these rabbits then showed an increase in responding within the first day of tone-alone extinction and across the first two to three days of extinction suggesting that the cues and the context may have reinstated at least part of the original memory before responding decreased with additional days of extinction [50, 51]. However, as few as one or two reminder trials in which the tone was once again paired with the US was sufficient to fully restore responding to the high levels seen at the end of acquisition [50] suggesting the rabbits had not forgotten the original association at all. Given the ability of a cholesterol diet to facilitate the acquisition of a task, one could argue that the cholesterol diet, rather than retard the memory of tone and air puff, may have improved the ability of the rabbit to learn something new—tone is not followed by air puff.

To understand and evaluate the relevance of the cholesterol-fed rabbit model to the cognitive deficits in Alzheimer's Disease, we need to understand how cholesterol specifically affects learning and memory. Although there is a great deal of research on the peripheral effects of a cholesterol diet, there is a relative lack of research on measurable behavioral indices such learning *and* memory. The need to look beyond cholesterol effects on response acquisition to effects on memory is very relevant to Alzheimer's Disease because one of the most devastating hallmarks of the disease is the loss of more recent memories—forgetting who your children are but not forgetting about your own childhood. The present results show that a cholesterol diet can indeed affect memory retention, but that it is a more subtle effect than found with studies on acquisition, suggesting a much more complicated, complex story, especially because it has to do with immediate versus long-term effects of the cholesterol diet.

It is clear that we have only begun to scratch the surface of cholesterol's effects on learning and memory. One of our most immediate tasks is to parse out the different peripheral and central effects of a cholesterol diet and how they might correlate with memory impairments. There are significant central as well as systemic consequences of a high-cholesterol diet. The central effects include, but are not limited to, compromise of the blood brain barrier [5, 8], increases in cholesterol metabolites such as 27-hydroxycholesterol [84], an elevation in inflammatory markers [85, 86], the accumulation of beta amyloid [6, 87], damage to the myelin sheath and axon [88], and changes in membrane cholesterol [78]. As noted earlier, the peripheral effects include a reduction in weight gain, increased serum cholesterol, inflammation and hepatotoxicity. The current data provide some suggestion that extinction is negatively correlated with these peripheral effects of a cholesterol diet. It remains to tease apart the differences between retention deficits and facilitated extinction and how cholesterol might affect each of them.

## 10. Conclusion

Manipulating cholesterol concentration for eight weeks following classical trace conditioning of the rabbit's nictitating membrane response retards long-term memory and the level of retardation is correlated with the level of cholesterol in the blood. Manipulating the duration of cholesterol for as little as four weeks also affects memory and depends upon when the manipulation takes place. The data support the idea that dietary cholesterol may retard long-term memory and begin to probe the nature of long-term memory, its consolidation, the nature of forgetting, and the role of the internal state of the animal plays during these important functions.
